# Population medical genetics: translating science to the community

**DOI:** 10.1590/1678-4685-GMB-2018-0096

**Published:** 2019-04-11

**Authors:** Roberto Giugliani, Fernanda Bender, Rowena Couto, Aline Bochernitsan, Ana Carolina Brusius-Facchin, Maira Burin, Tatiana Amorim, Angelina Xavier Acosta, Antônio Purificação, Sandra Leistner-Segal, Maria Luiza Saraiva-Pereira, Laura Bannach Jardim, Ursula Matte, Mariluce Riegel, Augusto César Cardoso-dos-Santos, Graziella Rodrigues, Marcelo Zagonel de Oliveira, Alice Tagliani-Ribeiro, Selia Heck, Vanusa Dresch, Lavínia Schuler-Faccini, Francyne Kubaski

**Affiliations:** 1 Medical Genetics Service, Hospital de Clínicas de Porto Alegre, Porto Alegre, RS, Brazil; 2 Department of Genetics and Molecular Biology, Universidade Federal do Rio Grande do Sul, Porto Alegre, RS, Brazil; 3 Instituto Nacional de Ciência e Tecnologia de Genética Médica Populacional (INaGeMP), Porto Alegre, RS, Brazil; 4 Postgraduate Program in Medicine: Medical Sciences Universidade Federal do Rio Grande do Sul, Porto Alegre, RS, Brazil; 5 APAE, Salvador, Brazil; 6 Escola Bahiana de Medicina e Saúde Pública, Salvador, BA, Brazil; 7 Fundação Oswaldo Cruz (FIOCRUZ), Salvador, BA, Brazil; 8 Department of Pediatrics, Universidade Federal da Bahia, Salvador, BA, Brazi; 9 Department of Biochemistry, ICBS, Universidade Federal do Rio Grande do Sul, Porto Alegre, RS, Brazil; 10 Genetics Identification Laboratory, Hospital de Clínicas de Porto Alegre, Porto Alegre, RS, Brazil; 11 Postgraduate Program in Genetics and Molecular Biology, Universidade Federal do Rio Grande do Sul, Porto Alegre, RS, Brazil; 12 Postgraduate Program in Biological Sciences: Biochemistry, Universidade Federal do Rio Grande do Sul, Porto Alegre, RS, Brazil; 13 Postgraduate Program in Celular and Molecular Biology, Universidade Federal do Rio Grande do Sul, Porto Alegre, RS, Brazil; 14 Department of Internal Medicine, Universidade Federal do Rio Grande do Sul, Porto Alegre, RS, Brazil; 15 Prefeitura Municipal de Cândido Godói, Candido Godói, RS, Brazil

**Keywords:** Population Medical Genetics, genetic clusters, founder effect, population isolates

## Abstract

Rare genetic disorders are currently in the spotlight due to the elevated number of different conditions and significant total number of affected patients. The study of these disorders is extremely helpful for the elucidation of physiological processes related with complex disorders. Isolated populations are instrumental for the study of genetic disorders, considering their homogeneity and high proportion of affected patients in a small geographic area. These favorable conditions lead to the creation of a new discipline, known as “population medical genetics”, which integrates medical genetics, population genetics, epidemiological genetics and community genetics. In order to develop practical activities in this new discipline, the National Institute of Population Medical Genetics (INaGeMP) was created in 2008 in Brazil. INaGeMP has developed several tools and funded numerous research activities. In this review, we highlight three successful projects developed in the first 10 years of INaGeMP activities (2008-2018): a newborn screening pilot study for MPS VI in Northeast Brazil, the study of Machado-Joseph disease in Brazilian families with Azorian ancestry, and the high twinning rate in a small town in southern Brazil. The results of these projects in terms of scientific output and contributions to the affected communities highlight the success and importance of INaGeMP.

## Introduction

The interest on rare genetic disorders has been increasing exponentially due to several factors: new genetic disorders are still being described; several individuals and families are affected by rare disorders requiring attention and public policies from governments; and genetic disorders are also a good model for elucidation of physiological processes, thus providing knowledge applicable to non-genetic disorders.

Population medical genetics (PMG) is the area of medical genetics that aims at the study and medical care of the population, and not of the individual or the family, as is the normal practice in clinical or medical genetics (Castilla, 2005). Based on this assumption, in 2008 the National Institute of Medical Population Genetics (INaGeMP) was created.

This Institute, works on the interface of Medical Genetics, Population Genetics, Epidemiology Genetics and Community Genetics, serving as a driver for activities focused on PMG.

Throughout the last decade (2008-2018), this initiative became a reference in the investigation of rare genetic disorders on population isolates with the creation of several tools for the study of genetic disorders and congenital anomalies, such as CENISO (National Statistics of Isolates) and CELAISO (Latin American Statistics of Isolates). Additionally, INaGeMP supported initiatives related to PMG, such as the Congenital Malformations Atlas (http://en.atlaseclamc.org/), the osteochondrodysplasias website (http://ocd.med.br/), the Latin American Collaborative Study on Congenital Malformations – ECLAMC (http://www.eclamc.org/), and the MPS Brazil Network (www.mps.ufrgs.br), among other projects.

INaGeMP is a state-of-the-art initiative that succeeded not only to provide benefits to local communities, but it also contributed to increase the knowledge on this area providing helpful information to the scientific community, governmental, and nongovernmental organizations. The headquarter is located at the Hospital de Clínicas de Porto Alegre (HCPA), with several associated institutions being part of the network: Universidade Federal do Rio de Janeiro (UFRJ), Fundação Oswaldo Cruz (FIOCRUZ), Universidade Federal do Rio Grande do Sul (UFRGS), Universidade Federal do Pará (UFPA), and Centro de Educación Médica e Investigaciones Clínicas Norberto Quirno (CEMIC, Buenos Aires, Argentina).

In addition, many collaborating institutions participate of INaGeMP, running specific projects. This review will focus on three of these projects: Newborn screening for Mucopolysaccharidosis VI in a community in the Northeast of Brazil; Machado-Joseph disease in Brazilian families with Azorian ancestry in the South of Brazil; high twinning rate in a small town close to the Argentinian border, in the very South of Brazil.

## Newborn screening program for Mucopolysaccharidosis VI

The incidence of MPS VI varies from 1:43,2361 to 1:1,505,461 births (Valayannopoulos *et al.*, 2010 [Bibr B56] [Bibr B57]). However, in the county of Monte Santo, located in the countryside of the state of Bahia, the frequency of the disease is 1:5,000 inhabitants (Costa-Motta *et al.*, 2011). The county has about 52,000 habitants with an average annual rate of 1,200 live births (https://www.ibge.gov.br/), and due to its remote geographical location and its inhabitants culture, endogamy is common in this population, resulting in a homogeneous genetic background for several generations. Thus, several genetic diseases have been diagnosed at this region, including MPS VI.

Thirteen cases of MPS VI were diagnosed until the current date at this county, all of them with the same genotype, p.His178Leu, in homozygosis (Costa-Motta *et al.*, 2011). Despite the high cost of treatment and of the challenges involved in providing this kind of therapy in a remote setting, the drug has been supplied by the Ministry of Health and adequate facilities for infusions were set up by the local health authorities. The analyses of family members of these patients showed 40% of heterozygosity. These results, together with the analysis of pedigrees, strongly suggest the occurrence of a founder effect in this region. The high relative incidence of the disease, the existence of an available treatment, and the evidences that early treatment with enzyme replacement therapy (ERT) has a positive impact on the prognosis of the disease (McGill *et al.*, 2010) lead to the proposal of introducing newborn screening for MPS VI in this region.

Newborn screening should be performed with a simple, viable and rapid technique in samples of ease collection and transport (even from long distances). The biochemical assay in dried blood spots (DBS) described by Chamoles *et al.* (2001) and adapted by Civallero *et al.* (2006) was adapted to reduced volumes of substrate and reagents for the assay of arylsulfatase B (ARSB) activity in microplates. However, a high proportion of false positives was observed due to the difficult collection and transportation conditions (remote location, high temperature, long distances).

**Figure 1:**
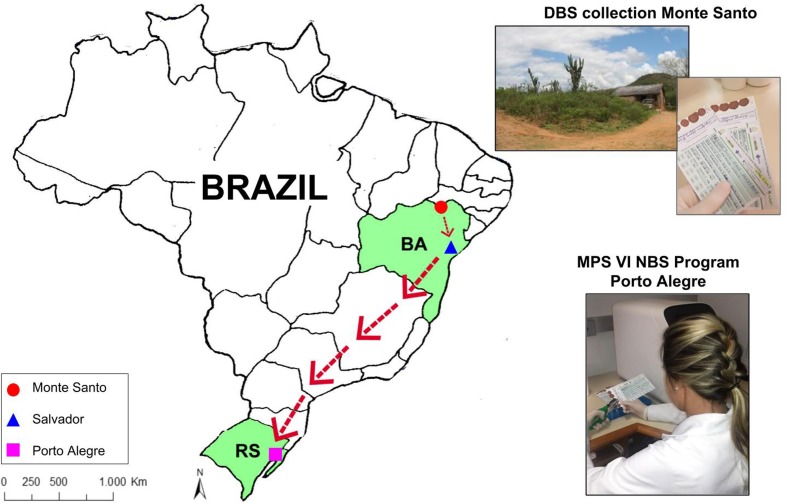
DBS collection from newborn of Monte Santo (Bahia state-BA), referred to the state reference center in Salvador (Bahia state-BA) and from there to the MPS VI Newborn Screening laboratory in Porto Alegre (Rio Grande do Sul state-RS).

In due course, INaGeMP worked to develop a test for the common mutation found in all patients in the region [p.His178Leu]. The analysis was developed with real-time PCR using custom designed TaqMan™ probes, at the Medical Genetics Service (MGS) of Hospital de Clínicas de Porto Alegre (HCPA). Samples from newborns from Monte Santo were collected and shipped to Porto Alegre by the Association of Parents and Friends of Exceptional (APAE) of Salvador, BA, from January 2011 to August 2017 (Figure 1).

Until now, DBS of 3,903 newborns have been analyzed and 67 (1.72%) were identified as heterozygotes for p.His178Leu. Although no homozygote patients have been identified yet, the number of heterozygotes enabled us to estimate a prevalence of approximately 1:16,000 live births. If confirmed, this frequency would be even higher than phenylketonuria (PKU), a disease included in the public newborn screening program in all states of Brazil. The identification of carriers allowed the identification of families at risk, enabling genetic counseling, carrier detection and prenatal diagnosis.

## Machado-Joseph disease, or spinocerebellar ataxia type 3

Machado Joseph disease (MJD) is a progressive and disabling autosomal dominant spinocerebellar ataxia that affects gait, speech, swallowing, and limb coordination. Since the first descriptions in 1972, MJD has been related to the people who migrated from the Azorean islands. After the discovery of the causal mutation, a translated CAG repeat expansion (CAGexp) in a gene now called *ATXN3*, molecular diagnosis enabled to define MJD as the most common spinocerebellar ataxia (SCA) worldwide (Saute and Jardim, 2015).

Researchers associated to INaGeMP have already been doing research on MJD since the nineties, when the first MJD families from Rio Grande do Sul were identified (Jardim *et al.*, 2001a,b,c). Preliminary evidence pointed to a founder effect in Rio Grande do Sul state (Jardim *et al.*, 2001a), and helped to improve phenotype characterization (Jardim *et al.*, 2001c), and to identify segregation distortion favoring the mutant allele (Jardim *et al.*, 2001b).

Since then, several approaches were followed to improve knowledge on this disease – from MJD epidemiology to selective forces, from modifier genes of MJD phenotype to biomarkers of disease progression, and from prospective studies to randomized clinical trials. This trajectory will be outlined below, especially in relation to PMG. A detailed comprehensive review covering these subjects can be found elsewhere (Saute and Jardim, 2015).

Without any modifier treatment, the need for natural history studies was imperative. The group published on survival estimates (Kieling *et al.*, 2007b) and developed the Neurological Examination Score for Spinocerebellar Ataxia (NESSCA) (Kieling *et al.*, 2008), a clinical scale used in our first longitudinal study about MJD progression (Kieling *et al.*, 2007a; Jardim *et al.*, 2010). Thanks to a very skilled statistical team, growth curves and Markovian chains were used in this pioneer study (Hauser *et al.*, 2009; Camey *et al.*, 2010). Later, a systematic review helped to determine the state of art of clinical scales in use (Saute *et al.*, 2012b [Bibr B45]). Already in the era of PMG, INaGeMP helped the next prospective cohort study that described the very bad progression rate of MJD starting during childhood (Donis *et al.*, 2016).

However, MJD progression as measured by clinical scales is slow, and biomarkers were required due to their potential as surrogate markers for future trials. The first potential biomarkers studied by our group included neurophysiology (Ghisolfi *et al.*, 2004), serum biomarkers related to glial and neuronal losses (Tort *et al.*, 2005 [Bibr B54]), loss of weight, and insulin (Saute *et al.*, 2011, 2012a). More recently, in a study sponsored by INaGeMP, it was possible to show that peripheral levels of eotaxin, a cytokine secreted by astrocytes, were increased in pre-clinical phases of the disease, and were reduced with disease progression, suggesting that astrocytes can have a protective role related to activation during the presymptomatic period (da Silva Carvalho *et al.*, 2016). Further studies on biomarkers also included neuroimaging (Klaes *et al.*, 2016) and oxidative stress (De Assis *et al.*, 2017). Trait biomarkers, i.e. modifier factors, were also investigated, such as the normal CAG repeat size at *ATXN2* (Jardim *et al.*, 2003). Potential associations between the heterozygous mutations of the glucocerebrosidase encoding gene (*GBA*) and Parkinsonism were also described in MJD (Siebert *et al.*, 2012), and between age at onset and modifiers, such as the methylation state of *ATXN3* (Emmel *et al.*, 2011) and the chaperone variants (Kuiper *et al.*, 2017).

A natural history study was crucial to estimate sample sizes for clinical trials, which are the next natural step. Following our previous experience with open trials (Monte *et al.*, 2003; Silva *et al.*, 2010), we embarked on the largest randomized, phase 2 clinical trial on safety and efficacy of a drug for MJD to date, and the outcomes showed that lithium was safe. By that time, knowledge on disease progression was limited to NESSCA, an instrument that covers all neurological deficits in SCA3/MJD, and due to that, it was chosen to be the primary endpoint of the efficacy. NESSCA remained unchanged after 48 weeks using lithium. However, secondary endpoints related to ataxic manifestations were significantly slowed down in the treated group when compared to placebo (Saute *et al.*, 2014). Motivated by these results, an *ad hoc* analysis was done in order to help planning future trials on MJD, in another analysis supported by INaGeMP (Saute *et al.*, 2015). Unfortunately, the role of other drugs on MJD progression remains unclear (Saute and Jardim, 2016).

The search for a modifier treatment for this autosomal dominant disease did not prevent us to focus on genetic counseling and on pre-symptomatic testing as primary prevention tools not only for familial planning, but also for reducing anxiety related to uncertainty. Decision making process on pre-symptomatic testing was the subject of two studies by our group (Rodrigues *et al.*, 2012; Schuler-Faccini *et al.*, 2014).

Nonetheless, in the light of PMG, probably some of the most interesting studies are those related to selective forces and to epidemiology of MJD.

**Figure 2:**
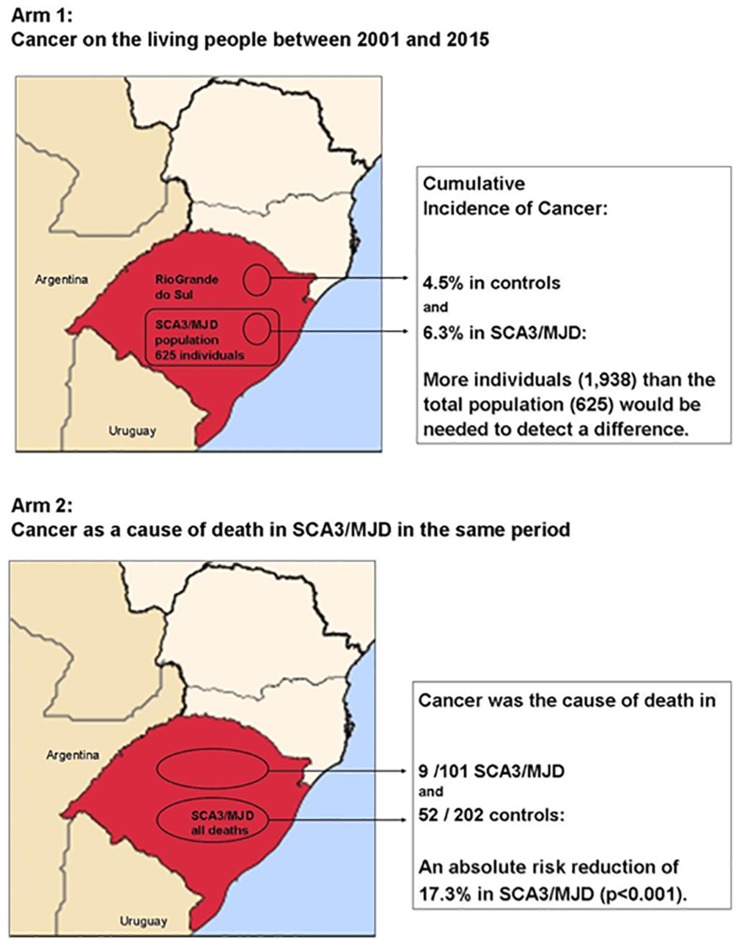
Cancer in SCA3/MJD versus general population of Rio Grande do Sul state, Brazil.

Anticipation phenomenon was a striking feature related to MJD transmission, noted before the discovery of a causal mutation (Saute and Jardim, 2015). Without counterweights, anticipation would lead to the removal of MJD from the population after few generations. Since there is no evidence suggesting that it was not happening, we then looked for positive selection forces related to the CAGexp at *ATXN3* in an antagonistic pleiotropism scenario. A population-based study on phenotype characterization showed that MJD carriers had more children, i.e. had an increased fitness, than unrelated and related controls (Prestes *et al.*, 2007). Psychosocial factors could hardly explain this finding: carriers had a higher number of offspring than non-carriers sibs even before the start of symptoms. Recently, a larger study deepened these evidences by genotypic information (Souza *et al.*, 2016). Based on this survey, we have estimated that MJD prevalence is 6:100,000 inhabitants in Rio Grande do Sul, where 625 symptomatic subjects were alive by 2015. These figures explain why MJD is by far the most frequent SCA in our region (Trott *et al.*, 2006; De Castilhos *et al.*, 2014). The recent study supported by INaGeMP (Souza *et al.*, 2016) endorsed our preliminary findings from 2001 that relate MJD to a segregation distortion favoring the mutant allele: genotyping of kindreds showed that 66% of sibs inherited the CAGexp. Therefore, at least two positive forces would increase MJD frequency in our population, increased fitness and segregation distortion, while just one negative force would be able to reduce it, the anticipation. To these formal genetic studies, we added another suggestion of antagonistic pleiotropism related to the presence of a CAGexp at *ATXN3*: a protective role against cancer (Souza *et al.*, 2017). Cumulative incidence of cancer among MJD carriers seemed to be lower than among non-carriers. Considering that MJD is a rare disease, population size prevented this study from confirming significant results. However, the frequency of cancer as a cause of death was significantly reduced among MJD carriers when compared to the local population (Figure 2). The meaning of these results supported by INAGEMP cannot be underestimated. Polyglutamine diseases (such as MJD) and cancer can be related in opposing cellular trends: increased cell death (as in neurodegenerative diseases) versus decreased death of neoplastic cells that occurs in cancer. Studying the association between MJD and reduced risk for cancer may benefit not only the understanding of evolutionary persistence of MJD in populations, but also research as well as drug discoveries for both neurodegeneration and malignancy.

## High twinning rate in Candido Godói, South of Brazil

Not every time a community draws attention due to a higher frequency of a genetic disorder, or a congenital anomaly. Here we present a case of a population in the South of Brazil that caught world attention for its high frequency of twin births. That would not be a problem if it were not for the fact that this high frequency of twinning was associated in the lay press and in social media as a product of experiments of an infamous Nazi medical doctor, Joseph Mengele (Camarasa, 1995). Although this would seem totally unfounded and scientifically unacceptable, it was not so for the population in question, a settlement of descendants of German immigrants whose majority arrived in Brazil during the nineteenth century. Pressed by local and international media, they reached out for expert advice to elucidate the “mystery” of the cause of high twinning.

Cândido Godói (CG; lat 27^o^ 57’ 07’; long 54^o^ 45’ 07’) is a small town in the South of Brazil with approximately 6,000 inhabitants. Our first visits to the community date back to 1994, when an investigator supported by the community visited and interviewed local families, reconstructed pedigrees and collected blood for future studies. The first conclusion was that the birth of twins was indeed increased in CG, estimated as 10% for the period 1990-1994, whereas for Brazil in general it was 1.8% (Matte *et al.*, 1996). Noteworthy, however, was the fact that the births of twins were not equally distributed throughout the municipality, but were mainly concentrated in one locality, named Linha São Pedro (LSP). It was also clear that the birth of twins was concentrated in a few, highly interconnected families.

It was not before 2008, with the beginning of INaGeMP that we had an opportunity to revisit the study of the families in CG, taking advantage of the huge advancement in molecular technology that occurred since 1994. At the same time, another book on the subject linking supposed experiments of the Nazi physician Joseph Mengele and the births of twins was published (Camarasa, 2008). These claims, of course, attracted wide media attention - the media had also evolved since1994, with the spread of the internet, and put the whole community of CG under scrutiny. Either they were collaborators in these experiments, or they were unaware victims subjected to pseudo-scientific research.

**Figure 3:**
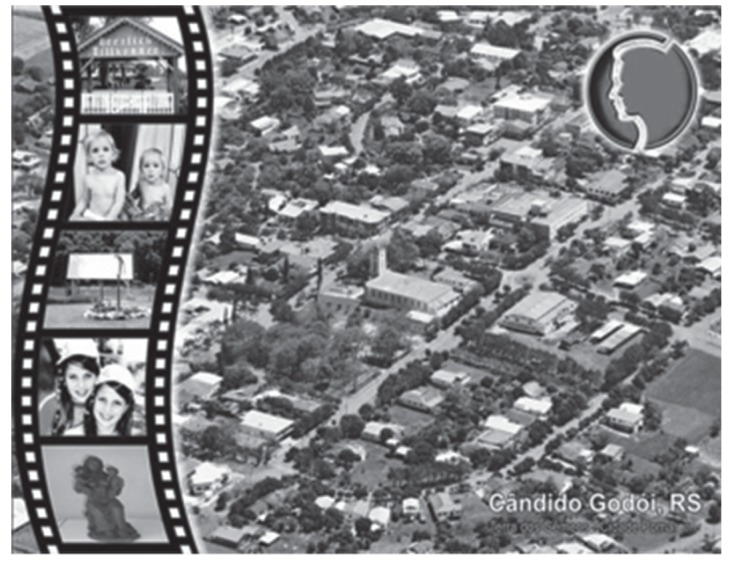
Sign in CG showing its own recognition as a “Twin Town”. Source: Prefeitura Municipal de Candido Godoi.

At this time, CG was proud of its title of “Twin’s Town”, with many local attractions on the subject, including a Twin’s Museum and a portico. Several signs in the city featured a symbol with two faces, representing the twins (Figure 3). Even a local festival was held every two years at LSP to gather twins from CG and elsewhere. Again, we travelled to CG, and with the help of the municipality, had meetings not only with the twins’ families, but also with other residents from CG. In these meetings we explained how we would work using scientific methodology, genetics principles, and multifactorial inheritance in order to engage them in a collaborative work with us. By this approach we tried to avoid the impression that they were again being subjected to scientific experiments and not having the full comprehension of what was happening. At the day that we settled to start the project, our team participated in the Sunday’s church service, and we were given the floor to announce the beginning of the field work in CG.

Our team was initially composed by geneticists (medical, population, and molecular genetics), geologists (geopositioning, water and soil analysis), a historian (family histories), a social scientist (baptism records, media reports) and a facilitator (to establish the contact and the logistic with the families and the field team), all working under the coordination of Ursula Matte (HCPA, the biologist who in 1994 had visited the community) and Lavinia Schuler-Faccini (HCPA/INaGeMP). Both the social scientist and the facilitator were from CG municipality.

Our basic assumption was that the high twinning rate in CG was the result of multifactorial inheritance, with underlying predisposition alleles concentrated due to a founder effect, associated with reproductive isolation. Therefore we designed a case-control study where cases were mothers of twins and controls were mothers of singletons, not being themselves part of a pair of twins. We also applied a detailed questionnaire to all families, to build pedigrees back to the oldest ancestor they had knowledge of and registering the presence of twins in the family. Dietary habits, weight, stature, obstetric and gynecologic information was also collected. Molecular analyses were performed looking for variations in candidate genes related to reproduction already described in the scientific literature. From these studies, we observed that the prevalence of the P72 allele of the *TP53* gene was higher in mothers of twins compared to mothers of singletons, suggesting a possible effect of this gene in the biology of twinning (Tagliani-Ribeiro *et al.*, 2012). This was the first study to associate the p53 pathway with twinning, and this finding was replicated in further studies in other populations (Huang *et al.*, 2015; Mardini *et al.*, 2017).

To better explore the hypothesis of the founder effect, we located geographically all residences where twins were born in CG, and also collected the surnames of all residents in CG as surrogates to estimate inbreeding. Our data supported the hypothesis of relative isolation of some communities in CG, particularly in LSP, where the twinning rate was higher, with low population dispersion and high inbreeding indexes (De Oliveira *et al.*, 2013).

But above all, we needed to provide a refutation to the “Mengele experiment” hypothesis. Therefore, we designed an epidemiological approach to test if there was a difference in the prevalence of twin births before the 1960’s and after. For this analysis, we tracked all baptism records from the municipal Catholic Church, as most inhabitants are of the Catholic religion. We surveyed 6,262 baptism records from 1927 to 2008 and tested for temporal trends and found no evidence of a spurt of twinning between or from the years 1964-1968, when Mengele was supposedly there (Tagliani-Ribeiro *et al.*, 2011). Moreover the use of surnames revealed that the mothers of twins had higher inbreeding coefficients than the mothers of singletons (De Oliveira *et al.*, 2013). Our findings were presented and discussed with the population again on a Sunday, in the church community saloon, before going for publication. At this day, pedigrees were printed and individually discussed with the members of the families interested.

This topic received a lot of media attention, both in our first visit (in 1994), as well as in the project’s second phase (from 2009 on). We limited ourselves on commenting published results only, and any request that involved contacting the community should be authorized directly by them. Even if sometimes we felt that cooperating with the media was positive for the community as a whole, we made it clear the decision was theirs. It was important that not only we had this attitude, but that this was perceived and understood by the community.

A consistent body of evidence strengthens the participation of genetic factors in the etiology of twin births, such as ethnic differences in twinning rates and intrafamilial predisposition for twinning (Hamamy *et al.*, 2004; Hoekstra *et al.*, 2008). Despite these evidences, only recently a few genes have being identified and implicated in the etiology of twin births, mainly DZ twins (Palmer *et al.*, 2006; Painter *et al.*, 2010; Tagliani-Ribeiro *et al.*, 2012; Mbarek *et al.*, 2016). The study of populations with naturally occurring high twinning rates may contribute to the study of the genetic factors associated with twinning, as shown here.

Besides reviewing the main results from the studies performed in CG, we detailed some particularities about this scientific investigation that involved the entire community. We believe that this type of work offers challenges different from those in which only individuals or families are affected by a certain phenotype. These challenges involve important issues, such as careful evaluation of ethical aspects in communication processes, to translate to the community the scientific process from hypothesis raising, methodology, operational procedures, and results. Moreover, the team tried to work WITH the community and not ON or FOR the community, to obtain their effective empowerment and autonomy after the research process is finished.

## Conclusion

After 10 years of activities (2008-2018), INaGeMP became an example of a successful initiative, supported with Brazilian public funding, that focuses on the study of clusters of genetic diseases and translates the results to practical health related actions in the affected communities, together with important contributions to science and creation of several tools (Costa-Motta *et al.*, 2011; Saute and Jardim, 2015; Donis *et al.*, 2016; da Silva Carvalho *et al.*, 2016).

INaGeMP has conducted projects related to several aspects of rare conditions, such as: MPS VI, genodermatosis, familial deafness, oral cleft, neural tube defects, thalidomide embryophathy, genetic susceptibility to Malaria and Leishmania, Machado-Joseph disease, genetic mutations associated with familial cancers. Other aspects studied are: elevated twinning rates, risks associated with exposition to nuclear energy, and congenital defects associated with chemical contamination in petrochemical areas.

We have also produced the “Four Legacies: Population Medical Genetics” documentary reflecting the challenges in dealing with genetic disorders and/or conditions in developing countries. The documentary is divided in four chapters: MPS VI, Machado Joseph disease, Tropical diseases and twinning rate (http://www.inagemp.bio.br/videos/quatro-herancas-genetica-medica-populacional/). The impact and importance of our work was recognized by comments in top ranked journals, such as Lancet (Fioravanti, 2014).

In summary, we developed an organization that became a template for other centers wishing to provide prevention, diagnosis, management, and research on genetic disorders, as well as centers aiming to work with local communities to address local needs. The expansion of this model to a higher number of communities, not only in Brazil but also in other Latin American and African countries was set as a goal for INaGeMP in the coming years.
